# Reducing the within-patient variability of breathing for radiotherapy delivery in conscious, unsedated cancer patients using a mechanical ventilator

**DOI:** 10.1259/bjr.20150741

**Published:** 2016-06

**Authors:** Michael J Parkes, Stuart Green, Andrea M Stevens, Sophia Parveen, Rebecca Stephens, Thomas H Clutton-Brock

**Affiliations:** ^1^ National Institute for Health Research (NIHR)/Wellcome Trust Birmingham Clinical Research Facility, University Hospitals Birmingham NHS Foundation Trust, Birmingham, UK; ^2^ School of Sport, Exercise and Rehabilitation Sciences, University of Birmingham, Birmingham, UK; ^3^ Hall Edwards Radiotherapy Research Group, Department of Medical Physics, University Hospitals Birmingham NHS Foundation Trust, Birmingham, UK; ^4^ Department of Oncology, University Hospitals Birmingham NHS Foundation Trust, Birmingham, UK; ^5^ Department Anaesthesia and Intensive Care Medicine, University Hospitals Birmingham NHS Foundation Trust, Birmingham, UK

## Abstract

**Objective:**

Variability in the breathing pattern of patients with cancer during radiotherapy requires mitigation, including enlargement of the planned treatment field, treatment gating and breathing guidance interventions. Here, we provide the first demonstration of how easy it is to mechanically ventilate patients with breast cancer while fully conscious and without sedation, and we quantify the resulting reduction in the variability of breathing.

**Methods:**

15 patients were trained for mechanical ventilation. Breathing was measured and the left breast anteroposterior displacement was measured using an Osiris surface-image mapping system (Qados Ltd, Sandhurst, UK).

**Results:**

Mechanical ventilation significantly reduced the within-breath variability of breathing frequency by 85% (*p* < 0.0001) and that of inflation volume by 29% (*p* < 0.006) when compared with their spontaneous breathing pattern. During mechanical ventilation, the mean amplitude of the left breast marker displacement was 5 ± 1 mm, the mean variability in its peak inflation position was 0.5 ± 0.1 mm and that in its trough inflation position was 0.4 ± 0.0 mm. Their mean drifts were not significantly different from 0 mm min^−1^ (peak drift was −0.1 ± 0.2 mm min^−1^ and trough drift was −0.3 ± 0.2 mm min^−1^). Patients had a normal resting mean systolic blood pressure (131 ± 5 mmHg) and mean heart rate [75 ± 2 beats per minute (bpm)] before mechanical ventilation. During mechanical ventilation, the mean blood pressure did not change significantly, mean heart rate fell by 2 bpm (*p* < 0.05) with pre-oxygenation and rose by only 4 bpm (*p* < 0.05) during pre-oxygenation with hypocapnia. No patients reported discomfort and all 15 patients were always willing to return to the laboratory on multiple occasions to continue the study.

**Conclusion:**

This simple technique for regularizing breathing may have important applications in radiotherapy.

**Advances in knowledge:**

Variations in the breathing pattern introduce major problems in imaging and radiotherapy planning and delivery and are currently addressed to only a limited extent by asking patients to breathe to auditory or visual guidelines. We provide the first demonstration that a completely different technique, of using a mechanical ventilator to take over the patients' breathing for them, is easy for patients who are conscious and unsedated and reduces the within-patient variability of breathing. This technique has potential advantages in radiotherapy over currently used breathing guidance interventions because it does not require any active participation from or feedback to the patient and is therefore worthy of further clinical evaluation.

## INTRODUCTION

Both the rate and depth of spontaneous breathing vary markedly,^1^ for a number of complex reasons including anxiety^2^ and the fact that as soon as subjects think about breathing, they voluntarily change it. Variations in breathing pattern require management in imaging and radiotherapy planning and delivery, not only for breast cancer but also potentially for all tumours in the thorax and abdomen. This management will be especially important to make the best use of the imminent introduction of MR guidance into radiotherapy.^3^ Koybasi et al^4^ report that for irregular breathing patterns, four-dimensional CT may inaccurately characterize tumour motion and location, with negative consequences particularly for treatment delivered with scanned proton beams. Furthermore, the correspondence between breathing motion assessed by four-dimensional CT at the planning stage and the actual motion during each daily treatment is so far measured only rarely in routine clinical practice. Specialized techniques such as CyberKnife (Accuray, Inc., Sunnyvale, CA) measure this correspondence well and other approaches to tracking are developing. Gating methodologies appear to be accurate but are technologically complex and increase the duration of treatment slots.

Currently, variations in breathing pattern can be addressed only to a limited extent, by asking patients to breathe to a metronome or to achieve inflation volumes within visible guidelines.^2,5^ But, all such techniques depend on how well patients respond to feedback and continue to comply with instructions.

Mechanical ventilation with positive pressure is universally used in general anaesthesia and imposes a completely regular pattern of breathing frequency and inflation volume on the patient for as long as required. This is also used under anaesthesia in younger paediatric patients for imaging and radiotherapy delivery. There is little awareness of the fact that mechanical ventilation can also be performed easily on patients who are conscious and unsedated and requires no feedback to or participation from the patient. We have over 15 years' experience of mechanically ventilating healthy volunteers and patients^6−9^ for periods of up to 1 h.

The work here on patients was originally undertaken for a different purpose (to use mechanical ventilation to achieve single prolonged breath-holds of more than 5 min^10^). During this, we found it easy to mechanically ventilate patients with breast cancer who are conscious and unsedated. The resulting regularization of their breathing pattern was striking. We therefore felt it important to demonstrate and measure here this regularization as a proof of concept and feasibility study, since this regularization may offer advantages for imaging and for the delivery of radiotherapy treatment.

## METHODS AND MATERIALS

Conduct of experiments followed the Declaration of Helsinki^11^ and approval of the local research ethics committee. Experiments were performed using our established techniques^9,10^ as described below. We studied 15 female patients undergoing radiotherapy, with 12 patients having recently undergone chemotherapy (the last treatment being within 26–180 days). They were aged 37–74 years, all of whom were non-smokers and none had respiratory, cardiovascular or neurological disease, diabetes or obesity.

**Figure 1.**
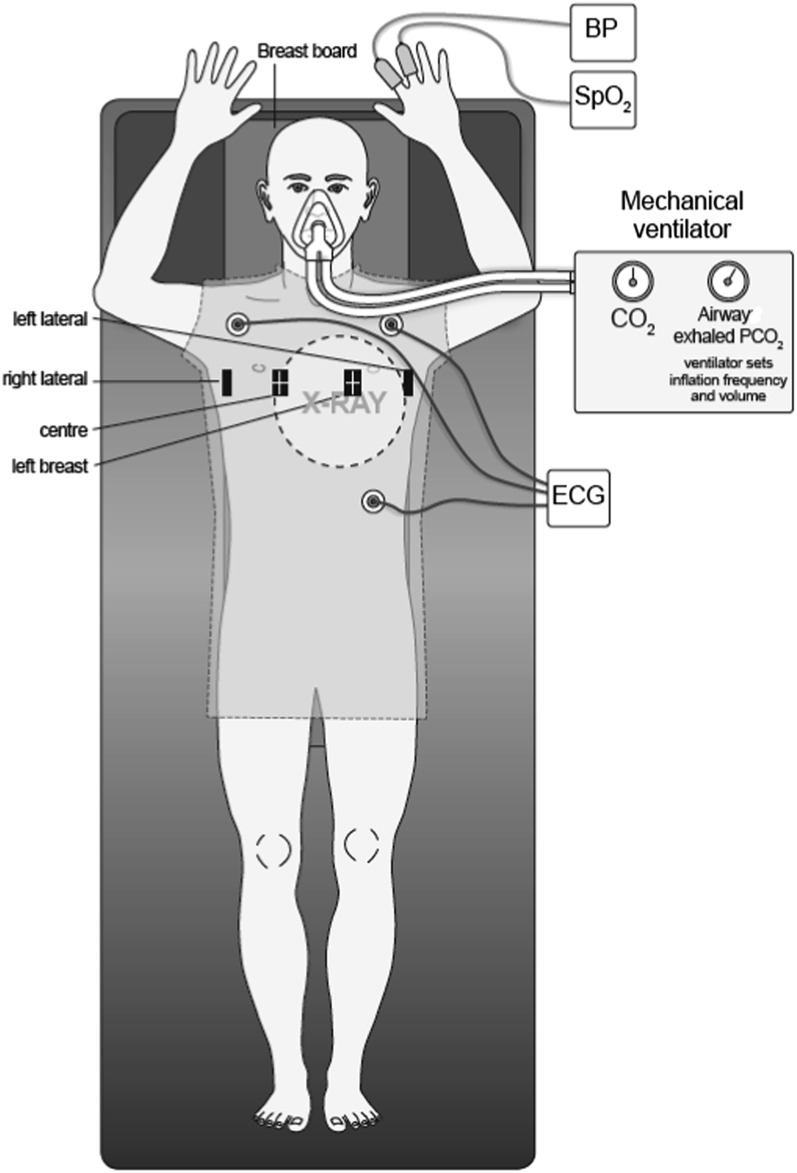
Equipment on the patient during mechanical ventilation in the simulator room. The figure shows a patient lying supine on a breast board, with blood pressure (BP) and oxygen saturation (SpO_2_) measured with non-invasive monitors on the fingers, a three-lead electrocardiogram (ECG) to measure heart rate and airway pressure and exhaled partial pressure of carbon dioxide (PCO_2_) measured in the face mask. The mechanical ventilator drives breathing via the face mask. The Osiris measures the movement of the chest with markers positioned at the centre of the chest, on the left breast and with right and left lateral markers oriented at 90° to the horizontal. The hypothetical X-ray target is indicated by the dotted circle. The Osiris surface-image mapping system was obtained from Qados Ltd, Sandhurst, UK.

Patients were given no medication before or during mechanical ventilation and they listened to music throughout. They lay at rest and were instrumented as indicated in Figure 1.

To connect patients with the mechanical ventilator, we used a disposable face mask, inline filter and flexible tubing. For safety, the mask contained a pressure transducer (Digitimer Ltd, Welwyn Garden City, UK) but no airflow meter. The gain of the transducer was set to produce a visible pressure wave during breathing [but without correcting to zero pressure, since the original purpose of these experiments did not require either zeroing of the pressure transducers to atmospheric pressure on each day or measurement of absolute pressure in international system of units (SI)]. Pressure here is therefore reported in arbitrary units (the same gain in every patient), and the absolute pressure was corrected to zero only after experiments were finished. The pressure wave here was then used to measure the regularity of breathing frequency and to estimate inflation volume (from the area of the pressure wave envelope).

To establish whether mechanical ventilation matched the metabolic rate of patients, we measured the partial pressure of carbon dioxide (PCO_2_) in their expired gas using an inline carbon dioxide (CO_2_) analyzer (Hewlett-Packard 78536A capnograph; Hewlett Packard, Palo Alto, CA).

While mechanical ventilation is so safe that the following measurements would not be necessary during routine radiotherapy, we also measured non-invasively the blood pressure [using a Finapres 2300 (Ohmeda, Englewood, CA) finger plethysmograph set at heart level], electrocardiogram and oxygen saturation [SpO_2_, Nellicor^™^ (Boulder, CA) finger pulse oximeter] of the patients as described previously.^6,8,12^

Patients were first taught to breathe spontaneously while connected to a Drager Evita 2 mechanical ventilator (Drager, Lubeck, Germany) on its assisted spontaneous breathing setting.^6−8^ This training was undertaken in a training laboratory in the Wellcome Trust Clinical Research Facility. Inspiration was made more comfortable by assisting with a small amount of pressure support (approximately 10 cm water = 10 mbar). This overcame the resistance of the connecting tubing and, by removing any sensation of inspiratory effort, ensured that breathing remained comfortable and effortless. All physiological data were sampled at 2 kHz, recorded and analyzed offline using a CED1401 data-acquisition system (Cambridge Electronics Design Ltd, Cambridge, UK).^10^

Patients were then taught to accept mechanical ventilation (the ventilator breathing for them) on its intermittent positive-pressure ventilation setting. For radiotherapy, mechanical ventilation can be undertaken with room air and at a wide range of frequencies and volumes to suit different patients and the particular needs in delivering personalized radiotherapy (Discussion section). But, for our original protocol, all patients here were ventilated in air or in 60% oxygen (to extend breath-hold duration) and at 16–17 breaths per minute. We set inflation volume at approximately 1.2 l (depending on patient body size^9,10^) such that mechanical ventilation exceeded the metabolic rate and end tidal partial pressure of carbon dioxide (PetCO_2_) fell to 20 mmHg. Although inflation volume can be read from the ventilator control screen, we could not access this signal electronically to measure the variability in volume for every breath and at this stage, the volume setting was not noted.

Finally, patients went to a simulator room in the Department of Radiotherapy of University Hospitals Birmingham NHS Foundation Trust containing the same equipment and an Osiris surface-image mapping system (Qados Ltd, Sandhurst, UK) that sampled at 3 Hz and reported marker position to within 0.1 mm. They listened to music, wore a gown or shirt and lay on a breast board under the simulator with their arms supported above their heads, to mimic every aspect of radiotherapy treatment.

Osiris markers were fixed on the gown in the following positions: right lateral, left lateral, chest centre and left breast. These enabled us to track continuously the movement of markers on the chest surface in the *x* (left–right), *y* (superior/head: inferior/toe) and *z* (anterior/ventral: posterior/dorsal) positions. We chose not to fix the markers directly on their skin to preserve patient modesty and to ensure they remained as relaxed as possible. Because our original goal was only to measure breast movement during breath-holding, the Osiris was not turned on during spontaneous breathing. It was turned on only at approximately the last minute of mechanical ventilation before breath-holding (and throughout breath-holding itself^10^) and at this point, the inflation volume setting of the ventilator was also noted.

### Numerical and statistical analysis of data

To analyze the breathing frequency and inflation volume data from each patient using Spike2 software (Cambridge Electronics Design Ltd), we compared (using a paired design) their approximately 5 min of spontaneously breathing air with their approximately last 5 min of the mechanical ventilation with pre-oxygenation and hypocapnia in the simulator room. We excluded the last two voluntary breaths preceding each breath-hold. We calculated instantaneous breathing parameters by identifying from the airway pressure wave the start and end of each inflation (with the difference = inspiratory time, measured in seconds), each period between the end of each inflation and the start of the next inflation (with the difference = expiratory time, in seconds) and the time difference between the start of successive breaths (whose reciprocal is instantaneous frequency measured as breaths per minute). We estimated inflation volume from the uncalibrated pressure waves by first mathematically zeroing the pressure waves and matching the gains (so measuring pressure in the same arbitrary units for each patient). Volume was then estimated by integrating the pressure wave during inspiratory time (and expressed as arbitrary units seconds).

Data were exported to Excel^®^ (Microsoft, Redmond, WA), where for each subject, we calculated their mean instantaneous breath frequency and inflation volume during spontaneous breathing and mechanical ventilation. We quantified the variability of breathing within in each patient by measuring the standard deviation of their instantaneous breathing frequency and volume during spontaneous breathing and mechanical ventilation (with each standard deviation expressed as a percentage of its mean value). We then calculated the mean percentage reduction in within-patient variability between spontaneous breathing and mechanical ventilation for all 15 patients.

To analyze the amplitude, variability and drift of displacement of the left breast surface marker in the anteroposterior (*z*) plane during mechanical ventilation, the Osiris data were exported into Excel and marker positions plotted against time for the approximately 1-min recording period. We measured the mean amplitude of left breast displacement for all ventilator breaths by measuring the difference between the peak and trough marker position for each breath, calculating the mean for all breaths in each patient and then the mean for all patients. We measured the mean variability in the peak and trough position by measuring the standard deviation of the peak and trough positions for all breaths in each subject and calculating their means for all subjects. We measured the mean drift over time of the peak and trough marker position by fitting a linear regression line in the form of *y* *=* *ax* *+* *b* to all peaks or to all troughs for each patient. If there is no drift, each slope value (*a*, measured as mm min^−1^) should be zero (and peak and trough slopes should be the same). We then calculated the mean peak and trough drift for all patients.

Statistical analysis was performed using PASW^®^ statistics v.18 (SPSS Inc., Chicago, IL), with paired comparisons within patients and unpaired comparisons with the data from our healthy subjects in Ref. 9, using analysis of variance for repeated comparisons or Student's *t*-test for single comparisons. Significance was taken at *p* < 0.05, and means are given with ±standard error of the mean.

## RESULTS

In all patients, training to breathe spontaneously through the ventilator and to accept being mechanically ventilated took no more than 10 min and was always completed in their first session. With our training regime, all 15 patients easily accepted breathing through a mask, being mechanically ventilated, remaining passive, not requiring any feedback, appearing comfortable and disinterested in what was going on around them. After 10 min of our training, all could be ventilated routinely for all subsequent sessions. In all patients, the time from being in a relaxed position to allowing the ventilator to take over their breathing was less than 1 min. Patients were sufficiently comfortable that sometimes they fell into a light sleep during mechanical ventilation, and this did not introduce any problems or disruption of their breathing pattern.

Patients were content before mechanical ventilation, as evidenced by their resting mean systolic blood pressure (131 ± 5 mmHg) and mean heart rate [75 ± 2 beats per minute (bpm)] being normal and not significantly different from those of our healthy untrained subjects,^9^ nor from those of our trained subjects in previous studies.^6−9^ They continued to be so during mechanical ventilation. This is evidenced by their having no significant rise in the systolic blood pressure and minor changes in the heart rate [mechanical ventilation with pre-oxygenation lowered the resting heart rate by 2 bpm (*p* < 0.05) and this with hypocapnia raised the resting heart rate by 4 bpm (*p* < 0.05)]. We found similarly small or no such changes in the heart rate and blood pressure in previous studies with mechanical ventilation in healthy subjects.^6−9^ Afterwards, patients reported no discomfort and were always willing to return to the laboratory on multiple occasions to continue the study.

Patients breathed spontaneously at a mean instantaneous frequency of 12 breaths per minute and with mean instantaneous inflation volume of 0.8 arbitrary volume units. We had chosen to mechanically ventilate patients at a mean frequency of 16–17 breaths per minute with a mean inflation volume of 2.4 arbitrary volume units.

**Figure 2.**
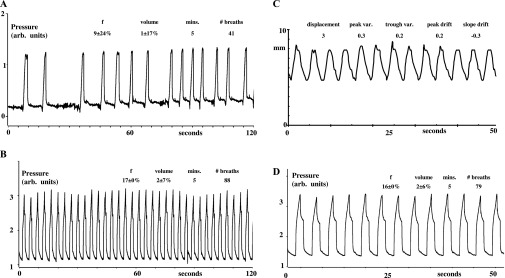
Mechanical ventilation regularizes breathing and breast movement in Patient 1. (a) Airway pressure during 2 min of spontaneous (irregular) breathing. Pressure measurements are uncalibrated [in arbitrary (arb.) units]. Spontaneous breaths in the mask cause negative pressure waves (downwards in the figure); but, for comfort, the ventilator then added approximately 10 cm water (approximately 10 mbar) of inspiratory assist, so each spontaneous breath continues as a positive pressure wave (upwards in the figure). For spontaneous breathing, the mean breathing frequency with its variability [±standard deviation (SD)%] within this patient in breaths per minute, inflation volume with its variability (±SD%) in arb. units seconds, the recording period duration (minutes) and number (#) of breaths during this period are indicated. (b) Airway pressure during 2 min of mechanical ventilation. The regular frequency and pressure amplitude indicate that the patient is passive (*i.e.* has allowed the ventilator to take over their breathing). For mechanical ventilation, the mean breathing frequency (f) with its variability (±SD%) within this patient in breaths per minute, inflation volume with its variability (±SD%) in arb. units seconds, the recording period duration (minutes) and number (#) of breaths during this period are indicated. (c) Left breast anteroposterior (ant.-post.) movement during 50 s of mechanical ventilation. The regular frequency and movement amplitude of the left breast confirms that the patient is passive and demonstrates how predictable is the breast movement during mechanical ventilation. The mean displacement (mm), variability (var) in peak and trough position (mm) and peak and trough drift (mm min^−1^) of the left breast marker are indicated. (d) Airway pressure during the same 50 s of mechanical ventilation as in (c). This demonstrates the correspondence between airway pressure and breast movement during mechanical ventilation and therefore that inflation volume is the same for each mechanically induced breath. For mechanical ventilation, the mean breathing frequency with its variability (±SD%) within this patient in breaths per minute, inflation volume with its variability (±SD%) in arb. units seconds, the recording period duration (minutes) and number (#) of breaths during this period are indicated.

**Figure 3.**
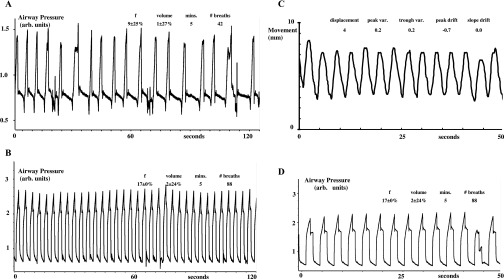
Mechanical ventilation regularizes breathing and breast movement in Patient 2. For descriptions on (a–d), refer to the legend of Figure 2.

**Figure 4.**
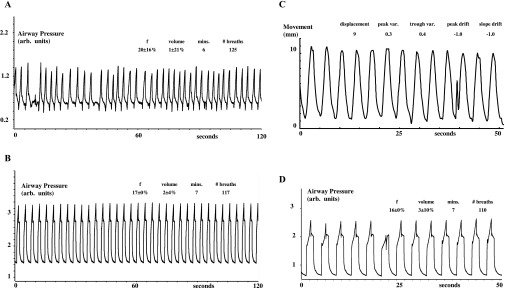
Mechanical ventilation regularizes breathing and breast movement in Patient 3. For descriptions on (a–d), refer to the legend of Figure 2.

**Figure 5.**
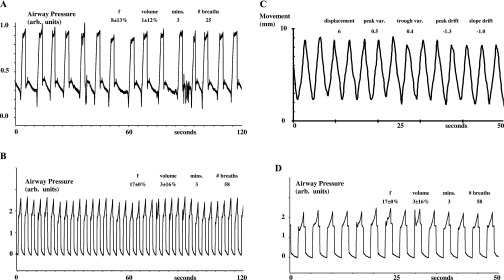
Mechanical ventilation regularizes breathing and breast movement in Patient 4. For descriptions on (a)–(d), refer to the legend of Figure 2.

Figures 2–5 show examples of breathing from four patients. Figures 2–5, section (a), show examples from four patients of the irregularities in spontaneous breathing (mainly in frequency) in a 2-min period. It also shows that different patients breathe spontaneously at different frequencies and with different irregularity patterns. Figures 2–5, section (b), show examples of how mechanical ventilation at 16–17 breaths per minute reduced the variability in both frequency and inflation volume in a 2-min period. Mechanical ventilation significantly reduced the mean within-patient variability of instantaneous breathing frequency by 85% (the mean within-breath standard deviation decreased from 19% to 3%, *p* < 0.0001, by Student's paired *t*-test) and that of instantaneous inflation volume by 29% (the mean within-breath standard deviation decreased from 24% to 12%, *p* < 0.007, by Student's paired *t*-test).

Figures 2–5, section (c), show examples of approximately 50 s of Osiris measurements of the left breast anteroposterior movement during mechanical ventilation [with their corresponding airway pressures shown in section (d)]. While the absolute displacement per breath of the left breast marker remains the same in each patient, the absolute displacement is different between patients (the mean being 5 ± 1 mm and the mean inflation volume setting on the ventilator being 1.2 ± 0.3 l, *n* = 15). This is because we had to set a different inflation volume for each patient, since the greater the metabolic rate of the patient, the greater the inflation volume necessary for ventilation at 16 breaths per minute to achieve a hypocapnia level of 20 mmHg.

Figures 2–5, section (c), show that the breast marker reached the same position (within approximately 1 mm) at the start and end of each inflation. Thus, the mean variability of peak position was 0.5 ± 0.1 mm (*n* = 15) and that of trough position was 0.4 ± 0.0 mm (*n* = 15). We measured no consistent drift over time in the position of the left breast marker at peak inflation and at peak deflation during mechanical ventilation. Thus, the mean drift (slope *vs* time) of mean peak position (−0.1 ± 0.2 mm min^−1^) and that of trough position (−0.3 ± 0.2 mm min^−1^) were not significantly different from either zero or each other.

## DISCUSSION

This study demonstrates in a small patient cohort that mechanical ventilation is easily achieved with patients who are unanaesthetized and unsedated. (Our experience is that patients, having been trained, accept being mechanically ventilated for up to about 1 h, after which some may become bored and restless.) Mechanical ventilation produces neither cardiovascular disturbances nor any discomfort nor negative responses that prevents them voluntarily returning to the laboratory on multiple occasions. We have used it successfully for many years in healthy subjects,^6−9^ but it is not widely used outside anaesthetic theatres. This is partly because few have seen its applications in healthy subjects, partly because of the common misconception that it is not possible in conscious subjects and partly because there is no inexpensive mechanical ventilator available at present for radiotherapy use. The purpose of this paper is to raise awareness that with suitable training, this technique is easily applicable to patients with cancer who are conscious and unsedated. We happen to have used it first on patients with breast cancer, but having found it so easy to use, it should be equally easy in patients with tumours at all other locations. Our next obvious patient groups for evaluation will be those with lung and liver tumours. It may have widespread application in radiotherapy, for both regularizing breathing and enabling single prolonged breath-holds of >5 min^10^ or to allow better synchronization of breathing patterns and radiotherapy delivery.

We show and quantify how variable is the frequency of spontaneous breathing and how mechanical ventilation reduces this variability. Since our experiments were carried out for another purpose, we did not deliberately measure inflation volume continuously during spontaneous breathing or mechanical ventilation (neither was the Osiris turned on until the last minute before breath-holding). Nevertheless, airway pressure is a reasonable estimate of inflation volume, because the relationship between inflation pressure and volume depends on chest compliance,^13^ and compliance can be taken as constant when subjects are passive. This is confirmed by the visible correspondence between inflation pressure and breast marker displacement when we did use the Osiris [Figures 2–5, sections (c) and (d)]. We also show and quantify how variable is the volume of spontaneous breathing and how mechanical ventilation reduces this variability too.

Our choice here of 16–17 breaths per minute at a relatively high inflation volume of approximately 1.2 l represents only one possible mechanical ventilation pattern, based on our experience as the most comfortable pattern to hyperventilate normal subjects to induce severe hypocapnia for other purposes.^6,9,10^ This choice resulted in a measured mean anteroposterior displacement of the left breast marker of 5 ± 1 mm. If we had chosen to use the mean spontaneous inflation volume of each patient (or even lower volumes at higher ventilation frequencies), then the mean displacement during mechanical ventilation could have been <2 mm per breath.

Mechanical ventilation can offer a wide range of inflation frequencies and volumes to suit patients and the particular needs in delivering personalized radiotherapy. Adult humans typically breathe spontaneously at approximately 12 breaths per minute, with an inflation volume of approximately 0.5 l^6,13,14^ (their product being a minute ventilation of approximately 6 l min^−1^), in order for breathing to match metabolic rate (and therefore to keep arterial blood gas levels constant). But, a minute ventilation of 6 l min^−1^ can be achieved by any suitable combination of slow deep or rapid shallow ventilation patterns. Conscious and unsedated subjects can also be ventilated at their own spontaneous frequency and volume.^6^ Mechanical ventilation, however, is inherently restful, not only because of the regular rhythm, but also because by taking over the work of the respiratory muscles (approximately 10% of metabolic rate^15,16^), it removes the effort of voluntary breathing.^6^ Thus, to satisfy ventilation now matching the 10% lower metabolic rate, patients would need to be mechanically ventilated only to achieve a minute ventilation of approximately 10% less (approximately 5.4 l min^−1^), using either a slightly smaller frequency and or volume. Moreover, the more relaxed they become, the lower their metabolic rate and hence the lower the required minute ventilation. The same minute ventilation of < 5.4 l min^−1^ could also be achieved by a wide range of slow deep or rapid shallow ventilation combinations outside the range that the patient might normally choose to use, depending on the comfort and requirements of personalized radiotherapy delivery. Furthermore, patients who are conscious may also be hyperventilated (a higher minute ventilation that exceeds metabolic rate and hence lowers the arterial partial pressure of carbon dioxide and raises that of oxygen), as was done here. Thus, even higher frequencies (at lower volumes) or lower frequencies (at higher volumes) are available for radiotherapy use, with the additional advantage that allowing the arterial partial pressure of carbon dioxide to fall reduces the patients' drives to breathe^6,7^ and hence makes it even less likely that they would want to override the ventilator. Further studies are now required to optimize ventilation parameters for personalized radiotherapy delivery.

In theory, mechanical ventilation should decrease the variability in breathing frequency and volume to zero, if patients are instructed to remain passive and never attempt voluntary breaths while being ventilated. Without such explicit instructions, patients can choose to assist or resist any imposed inflation, which will decrease or increase, respectively, the measured pressure wave. Furthermore, modern ventilators have the capability, if the patient tries hard enough, of allowing the patient to override temporarily the imposed inflation (after which, the ventilator then resets and restarts its pattern again). Both features are of great reassurance to patients who are conscious because they feel they can still be in control of their breathing if necessary and because they like the freedom to cough or sigh (or talk or laugh) during mechanical ventilation. But, because being ventilated is so restful, we found that once trained, patients who are conscious rarely assist, resist or reset the ventilator, as evidenced in Figures 2b–5c. Nevertheless, because our experiments had another purpose, we did not explicitly instruct patients never to take voluntary breaths while being ventilated. So, the 85% and 29% reductions in breathing frequency and volume variability we observed during mechanical ventilation underestimate the maximum reductions achievable in variability. We expect that under radiotherapy treatment conditions, patients will have no difficulty in remaining completely passive and disinterested and letting the ventilator breathe for them to produce a completely regular pattern for multiple periods of tens of minutes if explicitly required to do so.

In conclusion, mechanical ventilation has the advantage over all currently used breathing guidance interventions in radiotherapy that require patients to breathe to audible or visual guidance,^2,5^ because it does not require any active participation from or feedback to the patient (they can even fall asleep). It may offer particular advantages with the introduction of MR guidance into radiotherapy and for scanned particle therapy. It might even have application for older paediatric patients without the need for sedation. We look forward to formal clinical trials measuring the regularity of breathing in patients who are unsedated during imaging and radiotherapy treatment to establish what benefits this technique might offer.

## ACKNOWLEDGMENTS

We are most grateful to Cambridge Electronic Design Ltd, Dr David McIntyre and to Peter Nightingale for help with programming and statistical analysis, to Dr Tony Whitehouse and to Dr Una Martin and staff at the National Institute for Health Research/Wellcome Trust Birmingham Clinical Research Facility for help with patient training.

## CONFLICTS OF INTEREST

The views expressed are those of the authors and not necessarily those of the National Health Service, the National Institute for Health Research or the Department of Health. The authors Parkes, Green and Clutton-Brock have filed a patent for application of mechanical ventilation to radiotherapy.

## FUNDING

We are most grateful to the Queen Elizabeth Hospital Birmingham Charity who funded this project (Grant 17-3-645).
